# Population pharmacokinetic modelling to quantify the magnitude of drug-drug interactions between amlodipine and antiretroviral drugs

**DOI:** 10.1007/s00228-020-03060-2

**Published:** 2021-01-16

**Authors:** Perrine Courlet, Monia Guidi, Susana Alves Saldanha, Matthias Cavassini, Marcel Stoeckle, Thierry Buclin, Catia Marzolini, Laurent A. Decosterd, Chantal Csajka

**Affiliations:** 1 Service of Clinical Pharmacology, Lausanne University Hospital and University of Lausanne, Lausanne, Switzerland; 2 Centre for Research and Innovation in Clinical Pharmaceutical Sciences, Lausanne University Hospital and University of Lausanne, Lausanne, Switzerland; 3 Institute of Pharmaceutical Sciences of Western Switzerland, University of Geneva, University of Lausanne, Geneva, Switzerland; 4 Service of Infectious Diseases, Lausanne University Hospital and University of Lausanne, Lausanne, Switzerland; 5 Departments of Medicine and Clinical Research, University Hospital of Basel and University of Basel, Basel, Switzerland; 6 School of Pharmaceutical Sciences, University of Geneva, Geneva, Switzerland

**Keywords:** Amlodipine, Pharmacokinetics, Drug-drug interactions, HIV, NONMEM

## Abstract

**Purpose:**

Drug-drug interactions (DDIs) with antiretroviral drugs (ARVs) represent an important issue in elderly people living with HIV (PLWH). Amlodipine is a commonly prescribed antihypertensive drug metabolized by CYP3A4, thus predisposed to a risk of DDIs. Guidance on the management of DDIs is mostly based on theoretical considerations derived from coadministration with other CYP3A4 inhibitors. This study aimed at characterizing the magnitude of DDIs between amlodipine and ARV drugs in order to establish dosing recommendations.

**Methods:**

A population pharmacokinetic analysis was developed using non-linear mixed effect modelling (NONMEM) and included 163 amlodipine concentrations from 55 PLWH. Various structural and error models were compared to characterize optimally the concentration-time profile of amlodipine. Demographic and clinical characteristics as well as comedications were tested as potential influential covariates. Model-based simulations were performed to compare amlodipine exposure (i.e. area under the curve, AUC) between coadministered ARV drugs.

**Results:**

Amlodipine concentration-time profile was best described using a one-compartment model with first-order absorption and a lag-time. Amlodipine apparent clearance was influenced by both CYP3A4 inhibitors and efavirenz (CYP3A4 inducer). Model-based simulations revealed that amlodipine AUC increased by 96% when coadministered with CYP3A4 inhibitors, while efavirenz decreased drug exposure by 59%.

**Conclusion:**

Coadministered ARV drugs significantly impact amlodipine disposition in PLWH. Clinicians should adjust amlodipine dosage accordingly, by halving the dosage in PLWH receiving ARV with inhibitory properties (mainly ritonavir-boosted darunavir), whereas they should double amlodipine doses when coadministering it with efavirenz, under appropriate monitoring of clinical response and tolerance.

**Supplementary Information:**

The online version contains supplementary material available at 10.1007/s00228-020-03060-2.

## Introduction

The advent of antiretroviral therapies (ARVs) in the 1990s has revolutionized HIV care, now considered as a fairly manageable chronic condition. Meanwhile, the management of HIV infection is becoming more challenging, with an ageing HIV-infected population increasingly affected by physiological changes and age-related comorbidities. People living with HIV (PLWH) are predisposed to the risk of polypharmacy, thus increasing the burden of drug-drug interactions (DDIs). Indeed, ARV drugs are among therapeutic agents with the highest potential for DDIs, due to the frequent inhibition or induction of several cytochrome P450 (CYP) isoforms.

Despite its predisposition to be victim of DDIs with ARV drugs, amlodipine is a calcium channel blocker commonly prescribed in PLWH; this is probably explained by its rather large therapeutic margin. It is a molecule predominantly metabolized by CYP3A4/5 [1, 2], still with a lower hepatic extraction than other dihydropyridine calcium antagonists [3]. Nevertheless, it might be subject to potential DDIs with strong CYP3A4 inhibitors such as darunavir/ritonavir or with inducers such as efavirenz. However, the magnitude of DDIs with ARV drugs remained to be explored. Although the simple monitoring of blood pressure allows the evaluation of the clinical response to amlodipine, the knowledge of the magnitude of DDIs could guide drug dosage, taking into account the concomitant ARV treatment. The summary of product characteristics of amlodipine mentions an increase in amlodipine exposure (+ 60%) in the presence of diltiazem, a CYP3A4 inhibitor [4]. The label also indicates that a more pronounced increase is expected with other strong inhibitors like ritonavir, without further details on the magnitude of the interaction, neither with guidance on how to adjust amlodipine dosage. Moreover, DDIs between amlodipine and several antiviral agents for the treatment of chronic hepatitis C infection or first-generation ARV drug (e.g. ritonavir-boosted indinavir, which is no longer prescribed) have been evaluated using non-compartmental analyses [1, 5]. Authors demonstrated a 2-fold increase in amlodipine exposure when coadministered with these drugs, suggesting halving the dosage in individuals receiving such regimens. However, available data on DDIs were mostly collected in healthy young volunteers and therefore may not fully reflect the complex real-life situation of elderly PLWH.

Published population pharmacokinetic (PK) models have investigated amlodipine clearance in different populations (i.e. healthy volunteers, children, adolescents, patients living in nursing homes) and identified body weight, gender and age as the most important factors accounting for pharmacokinetic heterogeneity [6–10]. However, to our knowledge, no population PK models have been developed for amlodipine in PLWH.

The objectives of this study were thus to develop a population PK model of amlodipine in ageing PLWH and to perform model-based simulations to compare amlodipine exposure between various concomitantly prescribed ARV regimens, thus allowing the establishment of reliable dosage recommendations.

## Methods

### Study design and participants

Plasma samples were collected in PLWH followed up in Lausanne and Basel, in the framework of a prospective Swiss HIV Cohort Study project #815 designed to evaluate clinically significant DDIs between ARVs and frequently prescribed comedications. In addition, PLWH participating in the pharmacokinetic study NCT03515772 (registered in clinicaltrials.gov) contributed with intensive sampling. Study participants gave written informed consent before entering the study. The study protocol was reviewed and approved by the Ethics Committee of Vaud and northwest/central Switzerland (CER-VD 2018–00369).

Undetectable amlodipine plasma concentrations, suggestive of non-adherence to treatment, or samples with missing information about drug administration, sampling times or dosing schedule were excluded from the analysis. For all PLWH, age, bodyweight, gender, liver function tests (aspartate aminotransferase (AST), alanine aminotransferase (ALT), albumin), creatinine clearance (calculated with the Cockroft and Gault formula [11]), and comedications (HIV and non-HIV medications) were recorded.

### Plasma concentration determination

All blood samples were collected and centrifuged in EDTA-containing tubes. Plasma samples were stored at − 80 °C until batch analysis using an ultra-high-performance liquid chromatography tandem mass spectrometry (UHPLC–MS/MS) method [12]. Plasma samples underwent protein precipitation with methanol, followed by evaporation of the supernatant at room temperature under nitrogen. The lower limit of quantification was 0.3 ng/mL, well below trough concentrations commonly observed in clinical practice [9, 13, 14].

### Model-based pharmacokinetic analysis

#### Structural and error model

A population PK analysis was performed using non-linear mixed effect modelling (NONMEM v7.3, ICON Development Solutions, Ellicott City, MD, USA) to characterize amlodipine concentration-time profile in PLWH. PsN v4.2.0 was used for automation of model development and evaluation, Pirana v2.9.2 for structure model development and R v3.3.1 (Rstudio v.1.1.423) for data management, statistical analysis and graphical output [15, 16]. One- and two-compartment disposition models were compared, while evaluating various modelling options for the absorption phase: zero-, first-order or combined zero- and first-order absorption, with or without lag-time, or transit compartments models. Between-subject variability (BSV) was described by exponential errors following a log-normal distribution with mean zero and variance ω^2^, expressed ad coefficient of variation (CV). Several error models (i.e. proportional, additive and mixed) were compared to describe the residual variability.

#### Covariate model

First, the correlations between post hoc individual estimates of the PK parameters and the available biologically plausible covariates were inspected visually. Covariates considered as potentially influent were then sequentially included into the model using a stepwise insertion/deletion approach. ARV drugs were classified as strong CYP3A4 inhibitors (i.e. ritonavir-boosted darunavir, cobicistat-boosted darunavir, ritonavir-boosted atazanavir, cobicistat-boosted elvitegravir) or moderate inducers (i.e. efavirenz, etravirine), according to the lists published by the FDA [17]. The effect of the weaker CYP3A4 inducer nevirapine was also tested. Linear or non-linear functions were used as appropriate (categorical variables coded as 0 and 1 and continuous variables centred on their median values). Missing values for continuous covariates were imputed to the population median value.

#### Model selection and parameter estimation

Amlodipine concentration-time profiles were fitted using the first-order conditional estimation method with interaction (FOCEI). Discrimination between hierarchical models was based on the variation of the objective function value (ΔOFV, −2 log likelihood, approximate chi-square distribution) using the likelihood ratio test. For one additional parameter, a decrease of ΔOFV exceeding 3.84 (*p* < 0.05) or 6.63 (*p* < 0.01) was considered statistically significant during model building and backward deletion steps, respectively. Reliability of the results was evaluated using diagnostic plots, along with precision of pharmacokinetic parameters estimates and eta-shrinkage.

#### Model evaluation and assessment

The bootstrap method implemented in PsN was employed to validate the stability and performance of the final population PK model, using 2000 bootstrap samplings with replacement [15]. Median parameter values with their 95% confidence interval (CI_95%_) generated by bootstrap were compared with the original model estimates. The predictive performance of the final pharmacokinetic model was evaluated with the normalized prediction distribution errors (NPDEs). In addition, prediction-corrected visual predictive check (pcVPC) was performed.

#### Model-based simulations

Amlodipine maximum (C_max_) and trough concentrations (C_trough_), along with area under the curve from time 0 to 24 h (AUC_0–24_), were computed in 1000 simulated PLWH per different ARV regimen (CYP3A4 inhibitors, efavirenz or ARVs devoid of interaction potential with amlodipine). Average C_max_, C_trough_ and AUC_0–24_ between ARV groups were compared.

## Results

### Data

A total of 163 amlodipine concentrations were available from 55 PLWH, eight of whom participated in the PK study with rich sampling and provided 84 concentrations. PLWH in the SHCS#815 project provided a median (range) of one sample (1 to 3) while the median was 11 (8 to 11) for individuals included in the rich PK study. Amlodipine was administered at dosages ranging from 2.5 to 10 mg once daily (qd). Three PLWH received amlodipine 5 mg twice daily (bid). The measured plasma concentrations varied from 0.4 to 70 ng/mL. Steady state was assumed for all PLWH. The characteristics of the study population are presented in Table 1. Ritonavir-boosted darunavir was the most frequently prescribed ARV with CYP3A4 inhibitory properties.

**Table 1:** Demographic and clinical characteristics of PLWH included in the model development dataset

Patients’ characteristics (*N* = 55)	Median [IQR] or *n* (%)
Age (years)	61 [53–70]
Male sex	41 (75)
Body weight (kg)	79 [71–91]
Missing data	9 (16)
Systolic blood pressure (mmHg)	140 [130–150]
Missing data	9 (16)
Diastolic blood pressure (mmHg)	83 [75–93]
Missing data	9 (16)
ALT (IU/L)	27 [20–41]
Missing data	11 (20)
AST (IU/L)	26 [20–35]
Missing data	11 (20)
Albumin (g/L)	42 [41–44]
Missing data	11 (20)
Creatinine clearance (mL/min)	82 [50–101]
Missing data	17 (31)
Comedications (*N* = 163)	*n* (%)
CYP3A4 inhibitors	
Ritonavir-boosted darunavir	27 (17)
Cobicistat-boosted darunavir	1 (1)
Ritonavir-boosted atazanavir	1 (1)
Cobicistat-boosted elvitegravir	7 (4)
CYP3A4 inducers	
Efavirenz	7 (4)
Etravirine	18 (11)
Others	
Nevirapine	8 (5)
Rilpivirine	2 (1)
Dolutegravir	103 (63)
Raltegravir	8 (5)
Anti-hypertensive agents	137 (84)

### Structural, statistical and covariate models

A one-compartment model provided the best fit for amlodipine disposition, while a first-order process adequately described absorption. The addition of an absorption lag-time (ALAG) significantly improved the fit (∆OFV = -7.39, *p* = 0.007).

BSV needed to be assigned only on clearance, as the addition of BSV on the other parameters did not improve data description (∆OFV > -1.84, *p* > 0.17). The population estimates of the PK parameters with the base model were an absorption rate constant (k_a_) of 0.66 h^−1^, an ALAG of 0.86 h, a volume of distribution of 980 L and a clearance of 15.7 L/h, with a CV of 61% characterizing its BSV.

An additive error model adequately described the residual (intra-patient) variability. Univariate analyses revealed clear effects of the CYP3A4 inhibitors (∆OFV = − 20.9, *p* < 0.001) and of efavirenz (∆OFV = − 10.8, *p* = 0.001) on amlodipine elimination, with clearance decreasing by 49% and increasing by 40%, respectively. Both these covariates explained together 48% of the variance on clearance [18]. In contrast, we did not identify significant effects for age, sex, weight, albumin, AST, ALT and creatinine clearance on amlodipine disposition (∆OFV > − 1.1, *p* > 0.29). Finally, coadministration of ritonavir-boosted darunavir in two patients (13 amlodipine concentrations) receiving etravirine prevented the estimation of the effect of etravirine on amlodipine disposition.

### Model evaluation

**Fig. 1:**
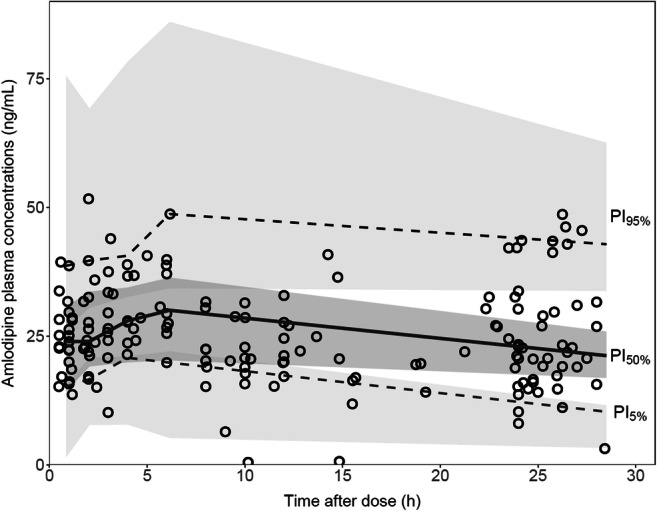
pcVPC of amlodipine final model with amlodipine prediction-corrected concentrations (open circles), median of the observed concentrations (solid line) with 90% prediction interval (dashed lines). Grey fields represent the model-based 90% confidence interval of the simulated median and PI_90__%_

The final PK parameter estimates are summarized in Table 2, along with their bootstrap estimations. Shrinkage was equal to 9% for BSV on clearance and to 15% for residual variability. All estimates lied within the bootstrap CI_95%_ and differed by less than 7% from the median bootstrap value, except for ka (17%), supporting the reliability of the model. Goodness of fit plots are presented in supplementary material 1. Normalized prediction distribution errors were not found to significantly differ from a normal distribution. As shown in Fig. 1, pcVPC confirmed the good predictive performance of the model.

**Table 2:** Final population parameter estimates of amlodipine with the bootstrap results. *k*_*a*_ first-order absorption rate constant, *ALAG* absorption lag-time, *CL* mean apparent amlodipine clearance, *V* mean apparent volume of distribution, *CI*_*95%*_ 95% confidence interval, *CYP* cytochrome P450, *CVs* coefficients of variation, *RSEs* relative standard errors defined as SE/estimate and expressed as percentages. CYP3A4 inhibitors included ritonavir-boosted darunavir, cobicistat-boosted darunavir, ritonavir-boosted atazanavir and cobicistat-boosted elvitegravir.

	Final pharmacokinetic model		Bootstrap (*n* = 2000 samples)	
Parameters	Estimate	RSE (%)	Median	CI _95%_
k_a_ (h^−1^)	0.69	25	0.80	0.44–2.15
ALAG (h)	0.87	28	0.90	0.24–2.41
V/*F* (L)	1000	16	985	777–1472
CL/*F* (L/h)	17.0	9	16.9	14.6–20.5
BSV_CL_ (CV%)	42	19	40	27–59
θ_CYP3A4 inhibitors_	−0.49	12	−0.49	−0.60 to −0.34
θ_efavirenz_	1.40	37	1.46	0.55–3.35
σ_add_ (ng/mL)	2.85	11	2.77	2.17–3.49

Final model: CL/*F* = 17.0 x (1–0.49 x CYP3A4 inhibitors) x (1 + 1.40 x efavirenz)

### Model-based simulations

Simulations showed a 96% increase and a 59% decrease of amlodipine AUC_0–24_ in patients taking the initial recommended dosage of 5 mg of amlodipine qd with CYP3A4 inhibitors and efavirenz, respectively, compared to amlodipine at the standard dosage alone.

**Fig. 2:**
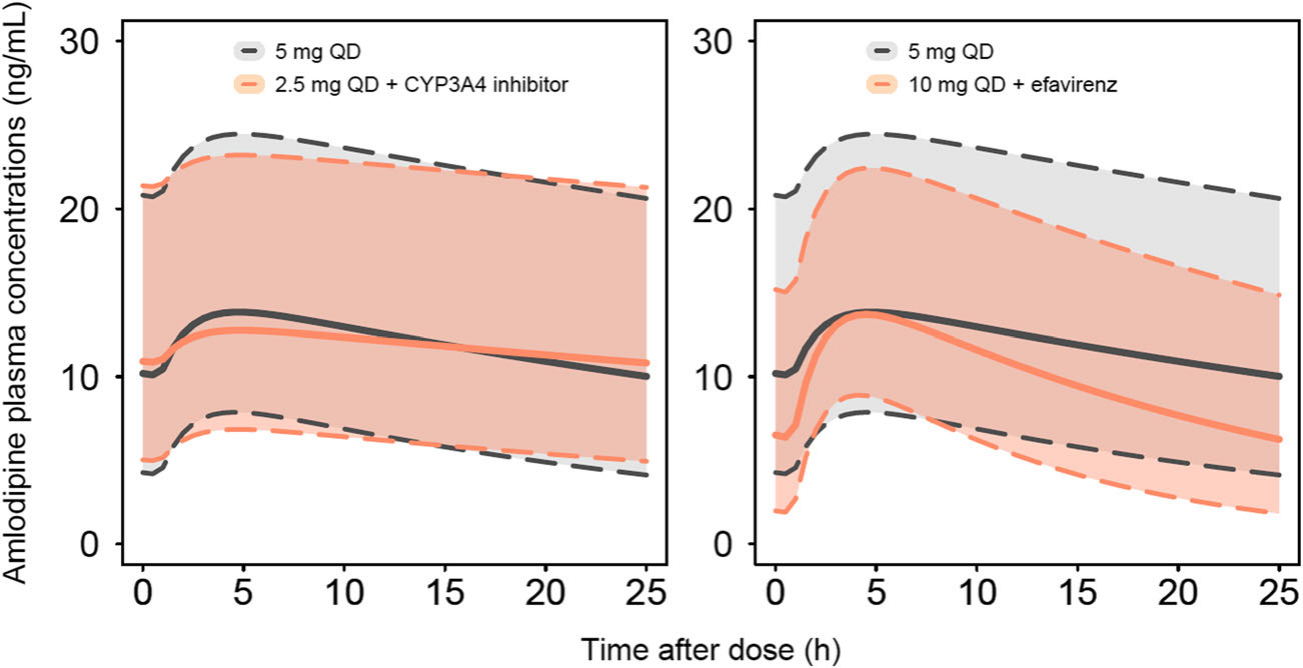
Simulated amlodipine plasma concentrations for dosage regimens of 2.5 mg qd with CYP3A4 inhibitors (i.e. ritonavir-boosted darunavir, cobicistat-boosted darunavir, ritonavir-boosted atazanavir, cobicistat-boosted elvitegravir) (left) or 10 mg qd with efavirenz (right), compared with standard dosage of 5 mg qd. Continuous line represent the population median prediction for standard (grey) and alternative regimens (orange), while shaded areas and dashed lines represent the 90% prediction interval based on 1000 simulated PLWH

Figure 2 compares amlodipine concentration-time profiles under the standard posology with alternative amlodipine dosage regimens in the presence of CYP3A4 inhibitors or efavirenz. The predicted concentration-time profile of 2.5 mg of amlodipine with CYP3A4 inhibitors almost entirely overlaps with the curve of 5 mg qd alone (8% and 2% decrease in C_max_ and AUC_0–24,_ respectively, while C_trough_ increases by 8% in the alternative vs standard regimen, Table 3). On the other hand, the dosage of 10 mg qd in the presence of efavirenz seems to provide slightly lower exposure (C_max_ increased by 1%, C_trough_ and AUC_0–24_ decreased by 38% and 17%, respectively, in the alternative vs standard regimen, Table 3). Increasing the dose to 15 mg qd in presence of efavirenz would result in a 51% and 25% increase in C_max_ and AUC, respectively, and a 7% decrease in C_trough_. A twice daily regimen of amlodipine when coadministered with efavirenz (i.e. 5 mg bid) would decrease C_trough_, C_max_ and AUC_0–24_ by 15%, 17% and 16%, respectively. These alternative dosage regimens (i.e. 15 mg qd and 5 mg bid) in presence of efavirenz are presented in supplementary materials 2 and 3.

**Table 3:** Summary of amlodipine PK parameters following several dosage regimens, in presence or absence of CYP3A4 inhibitors or efavirenz, derived from model-based simulations. PK values are presented as median (95% prediction interval). *C*_*max*_ maximal concentrations, *C*_*trough*_ residual concentrations (24 h after the last drug intake), *AUC*_*0-24*_ area under the concentration-time curve from 0 to 24 h calculated as dose/CL, *GMR* geometric mean ratio compared with the standard regimen of 5 mg qd

	Standard dosage of 5 mg qd	2.5 mg qd with CYP3A4 inhibitors	10 mg qd with efavirenz
C_max_ (ng/mL)	13.6 (7.3–29.0)	12.7 (6.2–27.8)	13.7 (8.4–26.4)
GMR		0.92	1.01
C_trough_ (ng/mL)	10.2 (3.6–25.5)	10.9 (4.3–26.0)	6.5 (1.5–19.0)
GMR		1.08	0.62
AUC_0–24_ (ng h/mL)	290.8 (129.3–658.4)	285.7 (127.0–646.8)	242.3 (107.8–548.7)
GMR		0.98	0.83

None of the PLWH receiving CYP3A4 inhibitors was concomitantly treated with efavirenz.

## Discussion

This study aimed to investigate the magnitude of DDIs between amlodipine and ARV drugs. For this purpose, the inhibitory or inducing potential of ARVs on amlodipine disposition was assessed in Swiss PLWH enrolled in two pharmacokinetic studies. The pharmacokinetic parameters of amlodipine alone are in good agreement with reported values, still with a lower apparent clearance in our analysis [6–9]. Several studies used a 2-compartment model to describe amlodipine concentration-time profile [7, 10, 19]. The sampling design of our study limited to 28 h after the last drug intake prevented us to capture adequately the prolonged terminal elimination phase of the drug [20]. However, the half-life calculated using our parameter estimates (40.8 h) was in accordance with the manufacturer’s data (35 to 50 h), and our model was thus considered adequate. Despite the important BSV, none of the tested demographic covariates showed any significant influence on the pharmacokinetics of amlodipine that would support dosage adjustment recommendations. In the present study however, we observe a 49% decrease in amlodipine clearance in case of coadministration of CYP3A4 inhibitors (mainly ritonavir-boosted darunavir). This result is in good agreement with a pharmacokinetic study conducted in healthy volunteers, where amlodipine exposure was increased by 90% when coadministered with ritonavir-boosted indinavir [1]. In addition, two physiologically based pharmacokinetic models reported a 2-fold increase in amlodipine AUC when coadministered with ritonavir [21, 22]. Two other studies showed a more pronounced increase in amlodipine exposure when coadministered with anti-HCV agents (2.6-fold increase in amlodipine AUC with ombitasvir/paritaprevir/ritonavir/dasabuvir and 2.8-fold increase with telaprevir [2, 23]). Accordingly, the “DDI-predictor” web-tool predicts an AUC ratio (AUC with interactor/AUC without interactor) of 2.7 based on several pharmacokinetic studies [24]. Using a similar approach, Stader et al. demonstrated a 90% increase in amlodipine exposure when coadministered with ritonavir [25]. Finally, the magnitude of DDI is not expected to differ when amlodipine is coadministered with ritonavir or cobicistat as these PK boosters are equally strong inhibitors of CYP3A4 that is responsible for amlodipine metabolism. However, differences in the magnitude of DDIs could occur for drugs whose metabolism involves CYP2B6, CYP2C9, CYP2C19, CYP1A2 or UGT since ritonavir induces these enzymes whereas cobicistat does not [26]. While the magnitude of the DDI between amlodipine and CYP3A4 inhibitors varies between studies, our results supports the proposed 50% reduction in amlodipine dosage in case of coadministration with ritonavir-boosted darunavir [27].

Although no upper tolerability threshold has been established for plasma concentrations, an increase in amlodipine exposure is not devoid of clinical consequences. Indeed, serious adverse events such as severe hypotension, oedema and bradycardia have been reported in PLWH receiving both calcium-channel blockers and ARVs with inhibitory potential [28–31]. This suggests caution when prescribing amlodipine in elderly PLWH receiving boosted ARVs, also considering pharmacokinetic and pharmacodynamic interactions with comedications and comorbidities.

To the best of our knowledge, coadministration of amlodipine and ARVs with enzyme inducing properties has not been studied until now. Our results demonstrate a 59% decrease in amlodipine exposure when coadministered with efavirenz. The lack of plasma concentrations from PLWH receiving etravirine alone prevented the estimation of the effect of etravirine on amlodipine PK. The inducing potency of etravirine is possibly lower than that of efavirenz, as observed, for example, on erlotinib and gefitinib (both CYP3A4 substrates) [32], although controversies persist about the respective inducing potential of etravirine and efavirenz [25, 32]. Pharmacokinetic-pharmacodynamic relationships have been established, indicating an impact of amlodipine plasma concentrations on antihypertensive effect [7, 9]. In case of treatment initiation, induction can take approximately 10 days to achieve its maximal effect, as reported for others CYP3A4 inducers [33]. However, the time course of CYP3A4 induction seems to be of limited clinical relevance in the case of amlodipine, prescribed for the long-term treatment of the chronic conditions of the patients included in our study. In addition, particular attention must be paid to elderly PLWH as they might be more sensitive to the effect of amlodipine given that the arterial baroreflex function is altered in elderly individuals [34]. Our results support the suggestion that amlodipine dosage should be doubled when coadministered with efavirenz, to reach a plasma exposure comparable to individuals not receiving interacting drugs, and therefore a similar antihypertensive effect. A bid regimen can also be proposed but lower adherence has been reported in PLWH receiving such an ARV regimen compared to a qd dosage [35]. In addition, PLWH from the Swiss HIV Cohort Study have lower adherence to their comedications in comparison to ARV [36]. Thus, a double dose in a qd regimen should be preferred over a bid regimen. This is also supported by the similar AUC_0–24_ (representing the global exposure) using a 10 mg qd regimen or a 5 mg bid regimen, both in presence of efavirenz. Obviously, amlodipine dosage decisions should rest on the global evaluation of the patient’s condition and response to treatment.

We have no observations of amlodipine exposure when coadministered with both CYP3A4 inhibitors and efavirenz. One PLWH included in the PK study with rich sampling received both ritonavir-boosted darunavir and etravirine, and had rather high amlodipine plasma concentrations. This indicates a predominance of the CYP3A4 inhibitory effect over the inducing potential of etravirine. This observation is in line with current knowledge on the coadministration of CYP3A4 inducers and inhibitors [37–40]. Regarding ARVs for example, a previously published study demonstrated a 3-fold increase in maraviroc exposure when coadministered with ritonavir-boosted darunavir and etravirine, compared to that obtained under maraviroc alone [41].

Limitations of the present work should be acknowledged. First, CYP3A4 inhibitors and efavirenz were the only covariates showing significant effects, while an effect of age, gender and body weight has been demonstrated in other population pharmacokinetics studies [6, 8, 9]. Yet, the limited number of patients and the sparse sampling schedule for most study data possibly compromise the statistical power of the study to reveal the effects of some covariates. In addition, the parsimonious one-compartment model may have affected the covariate selection [42]. Finally, the small number of PLWH receiving each ARV CYP3A4 inhibitor prevented us not only to establish an interaction model considering ARV drugs plasma concentrations but also to discriminate the effect of individual CYP3A4 inhibitors.

Despite these limitations, the information provided by our analysis on the magnitude of DDIs between amlodipine and ARVs is sufficiently robust to guide clinicians about initial drug dosage adjustments.

In conclusion, our results confirm the half-dosage proposed for amlodipine in case of coadministration of CYP3A4 inhibitors (mainly ritonavir-boosted darunavir), and suggest doubling the dose when amlodipine is coadministered with efavirenz.

## Supplementary Information

ESM 1 (DOCX 97 kb)

ESM 2 (PDF 14 kb)

ESM 3 (DOCX 18 kb)

## Data Availability

The data that support the findings of this study are available on request from the corresponding author. The data are not publicly available due to privacy or ethical restrictions.
