# Supplementary material for: Population pharmacokinetic modelling to quantify the magnitude of drug-drug interactions between amlodipine and antiretroviral drugs
Source: Eur J Clin Pharmacol. 2021 Jan 16;77(7):979–87. doi: 10.1007/s00228-020-03060-2 (PMC8184532; doi:10.1007/s00228-020-03060-2)

Supplementary 1: Goodness-of-fits plots for amlodipine final population PK model. a) Observed concentrations vs. individual predictions. b) Observed concentrations vs. population predictions. c) Conditional weighted residuals (CWRES) vs. population predictions. d) CWRES vs. time after the last drug intake.


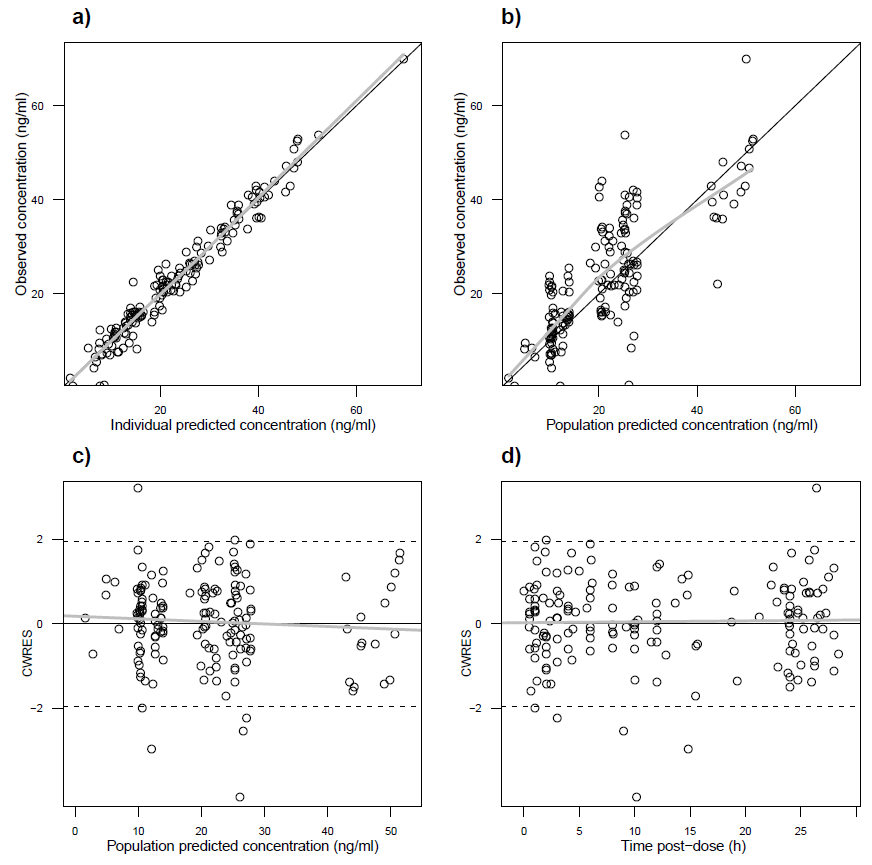

Supplement: Supplementary file 1 — (DOCX 97 kb) [file 228_2020_3060_MOESM1_ESM.docx]
