# Supplementary figures and images for: Population pharmacokinetic modelling to quantify the magnitude of drug-drug interactions between amlodipine and antiretroviral drugs
Source: Eur J Clin Pharmacol. 2021 Jan 16;77(7):979–87. doi: 10.1007/s00228-020-03060-2 (PMC8184532; doi:10.1007/s00228-020-03060-2)

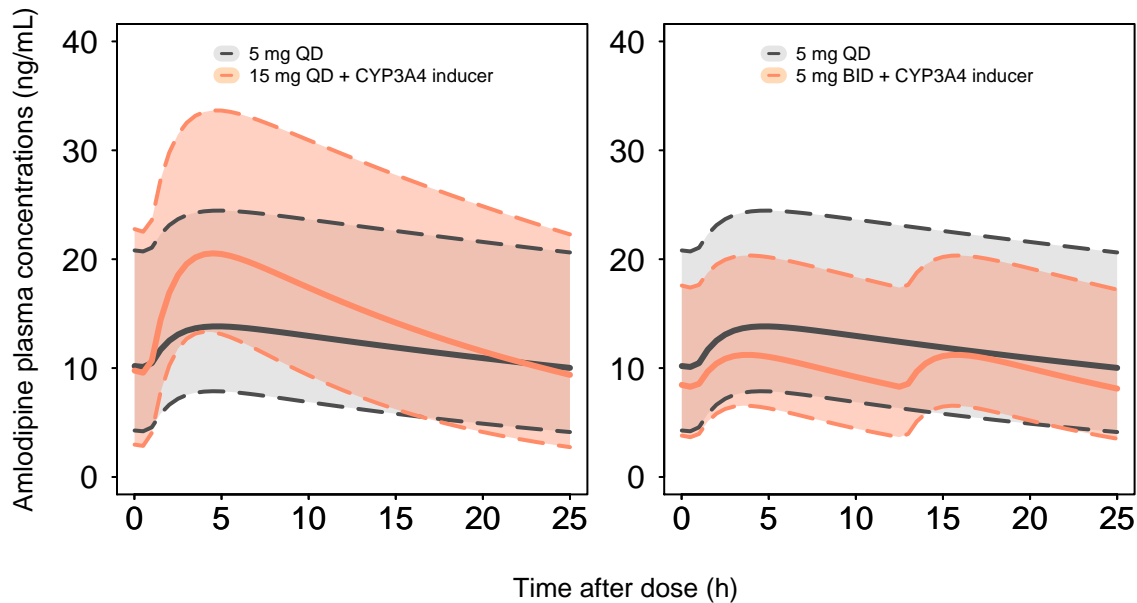

Supplement: Supplementary file 2 — (PDF 14 kb) [file 228_2020_3060_MOESM2_ESM.pdf]
