# Supplementary material for: Population pharmacokinetic modelling to quantify the magnitude of drug-drug interactions between amlodipine and antiretroviral drugs
Source: Eur J Clin Pharmacol. 2021 Jan 16;77(7):979–87. doi: 10.1007/s00228-020-03060-2 (PMC8184532; doi:10.1007/s00228-020-03060-2)
Supplement: Supplementary file 3 — (DOCX 18 kb) [file 228_2020_3060_MOESM3_ESM.docx]

Supplementary 3: Summary of amlodipine PK parameters following alternative dosage regimens, in presence or absence of efavirenz, derived from model-based simulations. PK values are presented as median (95% prediction interval).

C_max_: maximal concentrations, C_trough_: residual concentrations (24h or 12h after the last drug intake for a qd or a bid regimen, respectively), AUC_0-24_: area under the concentration-time curve from 0 to 24h, GMR: geometric mean ratio compared with the standard regimen of 5 mg QD.

|  | **Standard dosage of 5 mg qd** | **15 mg qd with efavirenz** | **5 mg bid with efavirenz** |
| --- | --- | --- | --- |
| C_max_ (ng/mL) | 13.6 (7.3 – 29.0) | 20.6 (12.5-39.6) | 11.2 (6.2-22.8) |
| GMR |  | 1.51 | 0.83 |
| C_trough_ (ng/mL) | 10.2 (3.6 – 25.5) | 9.8 (2.3-28.5) | 8.6 (3.4-20.1) |
| GMR |  | 0.93 | 0.85 |
| AUC_0-24_ (ng.h/mL) | 290.8 (129.3 – 658.4) | 363.5 (161.6 – 823.1) | 239.0 (117.0-517.8) |
| GMR |  | 1.25 | 0.84 |
